# The influence of ontogenetic diet variation on consumption rate estimates: a marine example

**DOI:** 10.1038/s41598-018-28479-7

**Published:** 2018-07-16

**Authors:** Christopher L. Lawson, Iain M. Suthers, James A. Smith, Hayden T. Schilling, John Stewart, Julian M. Hughes, Stephanie Brodie

**Affiliations:** 1 Evolution and Ecology Research Centre, School of Biological, Earth and Environmental Sciences, University of New South Wales, Sydney, NSW 2052 Australia; 2 Sydney Institute of Marine Science, Chowder Bay Road, Mosman, NSW 2088 Australia; 3 New South Wales Department of Primary Industries, Sydney Institute of Marine Science, Chowder Bay Road, Mosman, NSW 2088 Australia

## Abstract

Consumption rates are the foundation of trophic ecology, yet bioenergetics models used to estimate these rates can lack realism by not incorporating the ontogeny of diet. We constructed a bioenergetics model of a marine predatory fish (tailor, *Pomatomus saltatrix*) that incorporated high-resolution ontogenetic diet variation, and compared consumption estimates to those derived from typical bioenergetics models that do not consider ontogenetic diet variation. We found tailor consumption was over- or under-estimated by ~5–25% when only including the most common prey item. This error was due to a positive relationship between mean prey energy density and predator body size. Since high-resolution diet data isn’t always available, we also simulated how increasing dietary information progressively influenced consumption rate estimates. The greatest improvement in consumption rate estimates occurred when diet variation of 2–3 stanzas (1–2 juvenile stanzas, and adults) was included, with at least 5–6 most common prey types per stanza. We recommend increased emphasis on incorporating the ontogeny of diet and prey energy density in consumption rate estimates, especially for species with spatially segregated life stages or variable diets. A small-moderate increase in the resolution of dietary information can greatly benefit the accuracy of estimated consumption rates. We present a method of incorporating variable prey energy density into bioenergetics models.

## Introduction

Consumption is the basis of trophic ecology, and measuring it accurately is essential for modelling the impact of consumers and the trophodynamics of ecosystems^1^. Consumption rates change with ontogeny, and this change is typically expressed in bioenergetics models using allometric scaling, which defines the change in consumption rate with body size. However, allometric scaling is not the only factor influencing the ontogeny of consumption, and one frequently excluded factor is diet composition. Bioenergetics models can estimate a consumer’s energy requirements (in joules) but converting this to a consumption rate (in grams) requires information on the prey types consumed and their energy density. This data is lacking for many consumers and ecosystems, so bioenergetics models often use a single, common prey item of adults to represent the prey for all individuals of that species regardless of body size^2–16^.

Many consumers have diet shifts as they grow due to morphological changes and physiological needs, with ontogenetic diet variation observed in many freshwater^17,18^, estuarine^19,20^, and marine systems^21,22^. Without accounting for the ontogenetic variation in diet and its changing energy density, models that accurately estimate energy requirements (in joules) may be poor at estimating the consumption of prey (in grams) over the consumer’s lifetime. Incorporating diet variability into bioenergetics models and estimates of consumption is rare but it is important to acknowledge this as a source of uncertainty. This is difficult due to a lack of studies measuring the influence of ontogenetic diet variation on estimates of consumption.

Measuring the influence of diet variability is especially important for species with substantial ontogenetic variation, such as generalist predators, in which juveniles often target smaller and lower trophic level prey groups than adults^23^. Similarly, it is important to consider ontogenetic diet shifts for species with a spatially segregated juvenile phase. Along with higher consumption rates^24^, spatially segregated juveniles may consume different prey and reside in different habitats^25,26^, and therefore the predatory impact of juveniles may be directed elsewhere and in greater proportions than adults. To understand how consumption by multiple age groups of the same species may impact their respective ecosystems, size-specific consumption rates are needed that incorporate not just metabolic scaling, but the ontogeny of diet composition and its energy density.

The goal of this study was to quantify the change in consumption rate estimates when including ontogenetic diet and prey energy density variation in bioenergetics models. To achieve this, a preliminary goal was to develop a bioenergetics model parametrised by respirometry experiments to estimate consumption rates of a marine predatory fish (tailor, *Pomatomus saltatrix*). We used a high resolution dietary analysis of this species^27^, plus measured energy density of numerous prey types, to incorporate the predator’s ontogenetic diet variation into calculations of its consumption. This full model was compared to models using prey energy density from an individual prey item (a typical approach). Comprehensive diet data is not often available, so we also simulated how increasing dietary information (i.e. number of prey measured, number of consumer life stages or ‘stanzas’) progressively influenced consumption rate estimates, to identify the approximate resolution of dietary information required to achieve acceptable accuracy in consumption rate estimates.

## Results

### Metabolic rate

**Figure 1:**
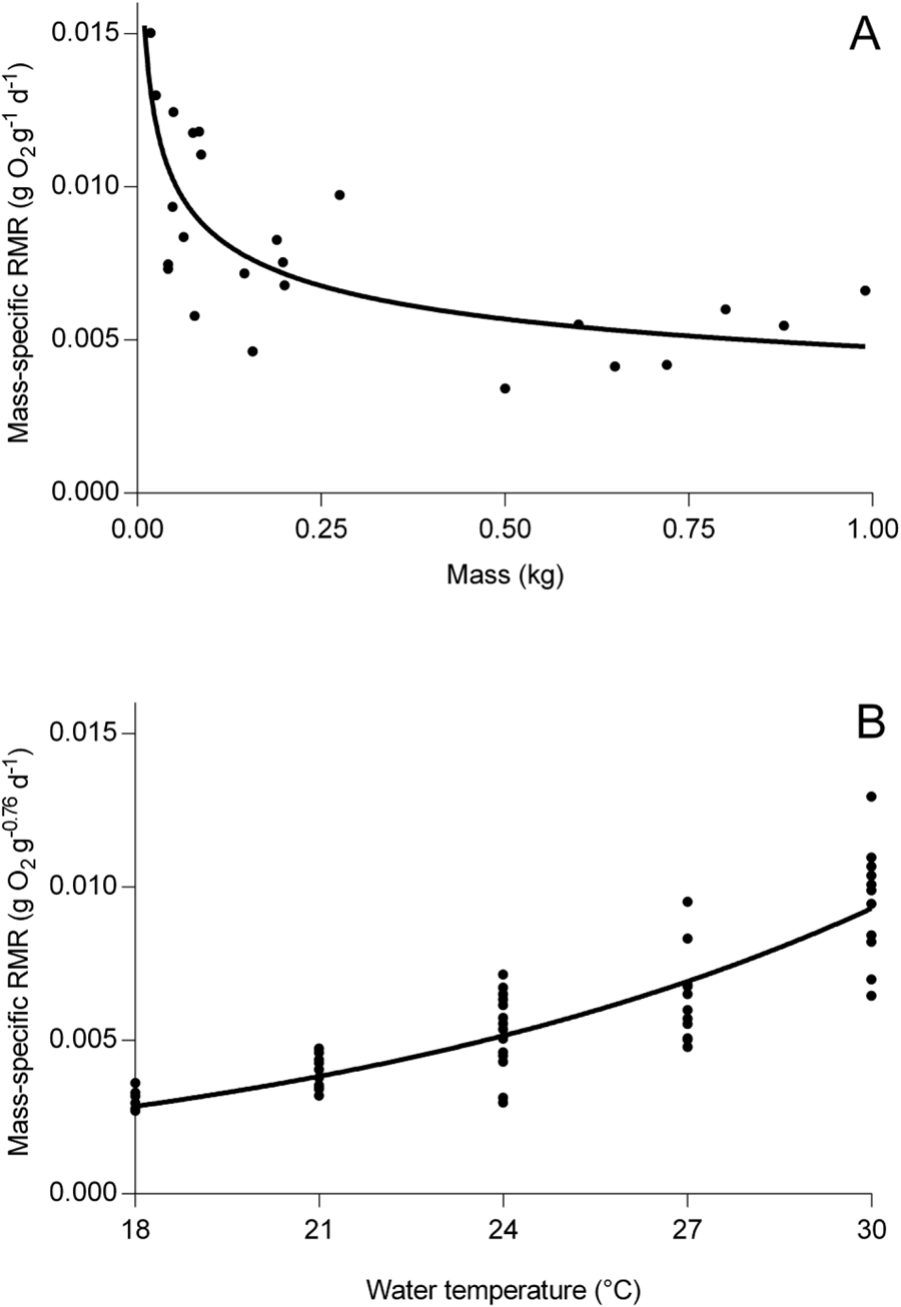
(**A**) Mass-specific resting metabolic rate (RMR) of tailor (n = 24) with increasing fish mass, at 24 °C. A linear regression of these data with both axes logged defines the parameter *R*_*A*_ (the intercept) and parameter *R*_*B*_ (the slope) from equation (3) (Table 2). (**B**) Mass-specific resting metabolic rate (RMR) of tailor (n = 12 per treatment) with increasing water temperature. The slope of logged values is the parameter *RQ* (equation (4), Table 2).

Respirometry experiments determined the mass- and temperature-dependent resting metabolic rate (RMR; gO_2_ g^−1^ d^−1^) of tailor. The mass-dependent respirometry experiment showed tailor RMR at 24 °C decreased with increasing body mass and was best described by a negative power curve (R^2^ = 0.54, n = 24; Fig. 1A). The temperature-dependent respirometry experiment showed tailor RMR increased exponentially with water temperature (R^2^ = 0.79, n = 61; Fig. 1B).

### Consumption rate estimates using ontogenetic diet information

Bomb calorimetry measured the energetic density of tailor and ten common prey items. *Sardinops sagax* (sardine) was the most energy-dense prey item (6.84 kJ g^−1^), while *Hyperlophus vittatus* (sandy sprat) was the least energy-dense fish prey (4.42 kJ g^−1^; Table 1). The only invertebrate tested, *Metapenaeus macleayi* (school prawn), had the lowest energy density of all prey items measured (3.84 kJ g^−1^; Table 1).

**Table 1:** Summary of mean values and standard error of the mean (s.e.m.) for energy density of representative tailor prey items measured by bomb calorimetry and used to calculate length-dependent mean energy density (*E*) of tailor prey. N = 10 for all species measured in this study.

Prey item	Energy Density (kJ g^−1^)	s.e.m	Source
*Pomatomus saltatrix*	7.06	0.42	This study
*Sardinops sagax*	6.84	0.33	This study
*Hyporhamphus regularis* ^a^	5.81	0.08	This study
*Trachurus novaezelandiae*	5.78	0.59	This study
*Acanthopagrus australis*	5.75	0.36	This study
*Scomber australasicus*	5.43	0.34	This study
*Liza argentea* ^b^	5.39	0.19	This study
*Gerres subfasciatus*	5.20	0.20	This study
*Sillago ciliata* ^c^	5.02	0.10	This study
*Hyperlophus vittatus* ^d^	4.24	0.19	This study
*Metapenaeus macleayi* ^e^	3.84	0.12	This study
Mysida	3.26	—	^83, 84^
Gobiidae	4.26	—	^85^
Atherinidae	4.23	—	^86^
Engraulidae	5.20	—	^87^
Polychaeta	3.06	—	^88^
Cephalopoda	3.90	—	^89– 92^
Decapoda (crabs)	2.63	—	^86^
Larval fish	4.18	—	^93^

^a^Used as a proxy for all members of Hemiramphidae. ^b^Used as a proxy for all members of Mugilidae. ^c^Used as a proxy for all members of Sillaginidae. ^d^Used as a proxy for all members of *Hyperlophus*. ^e^Used as a proxy for all members of Penaeidae.

**Figure 2:**
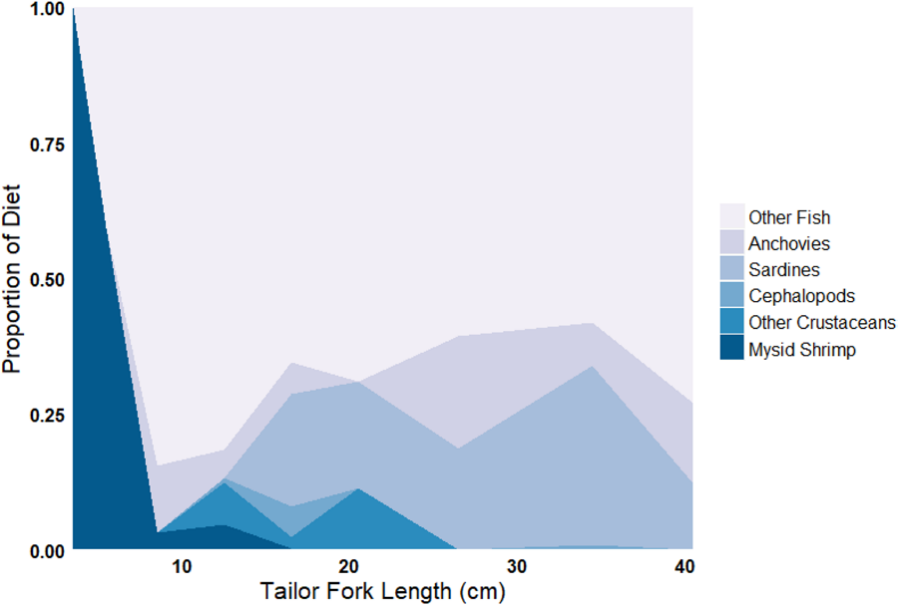
The proportion of common prey types consumed by tailor throughout their ontogeny n = 1437; adapted from^27^. The “Other Fish” category contains ~30 species of teleosts.

**Figure 3:**
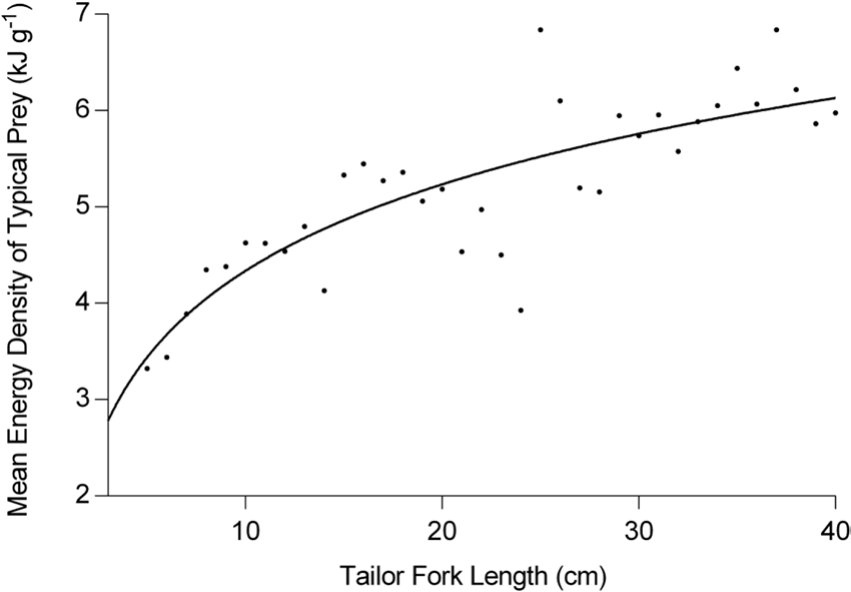
The mean energy density (*E*) of prey typically consumed by tailor (*E*, n = 1437 tailor stomach contents) throughout ontogeny (black dots represent mean values of 1 cm tailor size classes). The solid black line is the fitted curve described by equation (6).

**Figure 4:**
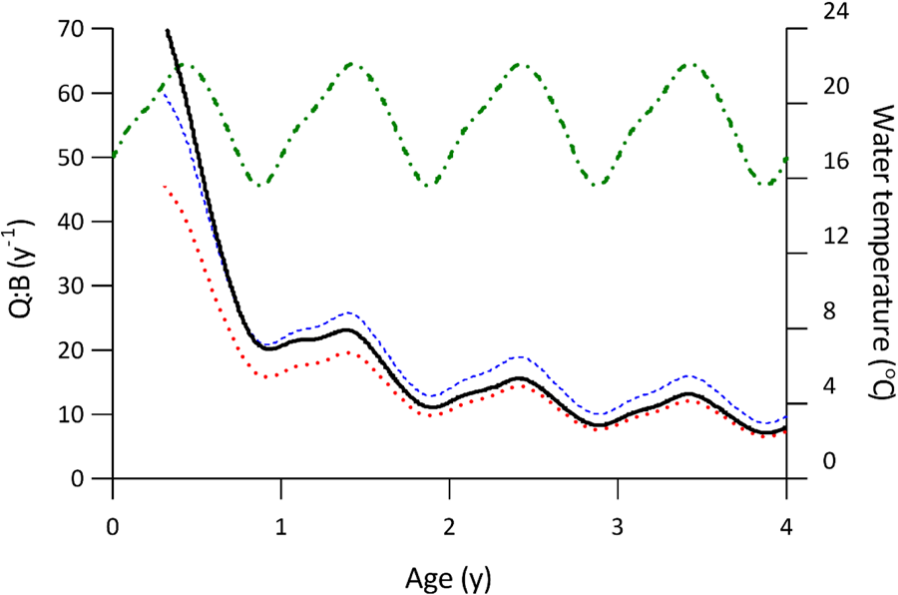
Modelled consumption: biomass ratios (Q:B) of tailor calculated using different prey compositions. A variable prey energy density (solid black), a constant prey energy density based on a 100% sardine diet (high energy content; dotted red), and a constant prey energy density based on a 100% anchovy diet (low energy content; dashed blue). Environmental water temperature is overlayed (dash-dot green).

The diet of tailor changed with ontogeny from a predominately invertebrate-based diet to a largely piscivorous diet (Fig. 2)^27^, with the mean energy density of typical prey species increasing asymptotically with predator size (Fig. 3). Juvenile tailor exhibited significantly higher mass-specific consumption than adults (Fig. 4). At one year of age, tailor consumed 5.7% of their body weight in prey daily, or an annual Q:B of 20.6 (Fig. 4). After 4 years, the daily consumption stabilised at 1.5–2.8% body weight (mean 2.1%), or a Q:B of 5.5–10.1 (mean 7.7), with cyclical variation caused by seasonal fluctuations in the mean daily water temperature of the study area, Sydney Harbour Australia (Fig. 4).

### Influence of ontogenetic diet information

In comparison with our bioenergetics model that included size-structured diet information, consumption rate estimates that included only the energy density of the most common prey (sardine, high energy density) underestimated tailor consumption (g) by 26.7% in their first year, and by 8.4% over their lifetime (7 years). Using the second most common prey (anchovy, low energy density) underestimated tailor consumption in their first year by 3.6% but overestimated lifetime consumption by 20.4%. In terms of mass-specific consumption rates, only including the energy density of sardines underestimated the Q:B of a 1 year old fish by 20.1% and of a 4 year old fish (when Q:B stabilises) by 6.6%, while only including anchovy energy density overestimated the Q:B of a 1 year old fish by 5.1% and of a 4 year old fish by 22.8% (Fig. 4).

### How many prey types, how many stanzas

**Figure 5:**
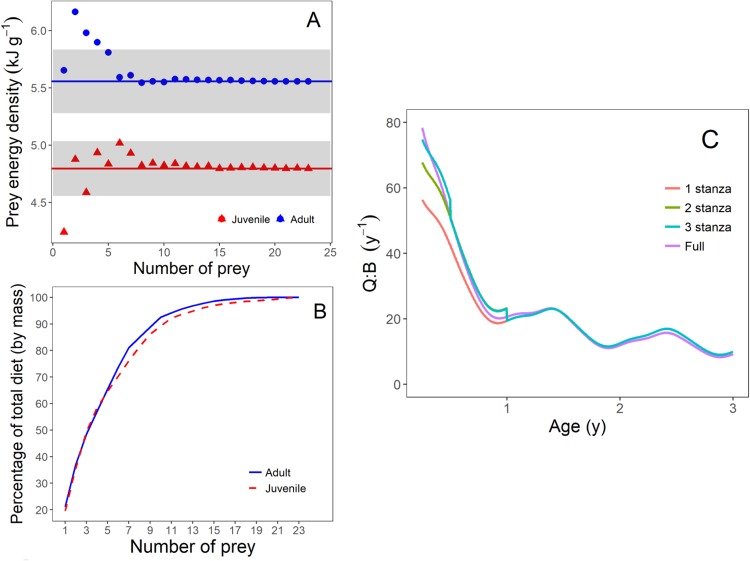
(**A**) The variation in mean prey energy density (kJ g^−1^) against prey number. The weighted mean energy density for juveniles and adults was calculated with the most common prey items added sequentially. Red triangles show juveniles (0–1 y), blue circles show adults (>1 y). Horizontal lines represent the actual weighted mean for each stage (i.e. the weighted mean when all prey items are included). Grey bands show ±5% from actual weighted mean for each stage. (**B**) The contribution (% by mass) of prey types to adult (full line) and juvenile (dashed line) tailor diet, illustrated as accumulation plots. C) The calculated relative consumption (Q:B, y^−1^) as the number of prey stanzas is increased (1–3), compared to the full model. Each stanza represents the age class for which a constant prey energy density is calculated (from the 5 most common prey types per stanza). The lines of the 1, 2, and 3 stanza simulations >1-year overlay. Compared with our full model, mean lifetime Q:B was underestimated by 11% using one stanza, 4% using two stanzas, and 1% using three stanzas. The x-axis is truncated at 0.25 and 3 years for clarity.

We conducted two simulations to identify the approximate resolution of dietary information required to achieve acceptable accuracy in consumption rate estimates. The first simulation revealed the number of most common prey items to include in our bioenergetics model to reach a reasonable estimate of true energy density. The simulation showed that for both juveniles and adults, 5–6 prey items were needed for the mean energy density to be within 5% of the ‘true’ mean (measured from all prey items) and to approach it monotonically (Fig. 5A). In both juveniles and adults, these five most common prey items accounted for 65% of the total diet (by mass; Fig. 5B).

The second simulation showed the influence of partitioning lifetime consumption into discrete stanzas. In general, the consumption rate error decreased and approached the full model when more stanzas were used. Compared to our full model, a single stanza using the weighted mean energy density of the five most common prey types underestimated mean juvenile Q:B by 28.9%, but this error declined when two (14.5%) and three (8.5%) stanzas were used (Fig. 5C). Mean lifetime Q:B was underestimated by 10.9% using one stanza, 4.0% using two stanzas, and 1.1% using three stanzas (Fig. 5C). In terms of absolute consumption (as opposed to relative consumption measured as Q:B), a single stanza using the weighted mean energy density of the five most common prey types overestimated total lifetime consumption by 7.7%, whereas total consumption was overestimated by 8.0% when two or three stanzas were used. Total juvenile consumption was underestimated by 13.7% when a single stanza was used but overestimated when two (3.6%) and three (5.3%) stanzas were used.

## Discussion

Robust consumption rate estimates of consumers are vital for understanding trophic ecology and predator-prey interactions. This is especially true for managing exploited fish populations or species that migrate across different habitats or ecosystems. By constructing a bioenergetics model that included ontogenetic diet variation of a marine predator, we demonstrated that variation in energy density of prey sources is an important component that can substantially alter estimated consumption rates. By simplifying diet information when estimating consumption (including mean ‘lifetime’ consumption), acceptable levels of accuracy were achieved (within 1–4% of full model) when mean energy density was calculated for 2–3 age stanzas using information for the five most common prey types per stanza. Although the resolution required will be somewhat species-specific, it is likely this level is an appropriate starting point for many predatory fish.

### The importance of ontogenetic diet information

Assuming a constant prey energy density throughout the life of a consumer overlooks key aspects of ontogeny, yet a lack of comprehensive dietary information for many species means this assumption must be frequently made in bioenergetics models. The models we present here show that incorporating consumer size-dependent prey energy density (based on detailed dietary information) is important for improving the accuracy of consumption estimates. It is common in bioenergetics models to use the energy density of only the most common prey item of adults, and our results showed that doing this (using sardine) underestimated the consumption rate of juvenile tailor by ~27%, and by ~8% over their lifetime (7 years), compared with our full model that incorporated predator size-dependent prey energy density. When the second most common prey item of adults (anchovy) was used, the consumption by juveniles was coincidentally similar to the full model but the consumption: biomass ratio (Q:B) of adult tailor was even further overestimated (by ~23%).

Without a size-dependant diet analysis, it is reasonable to assume that either of the two most common prey species could be used as a “one size fits all” value of prey energy density. Yet a large amount of variation in energy density can exist between the most common prey items and selecting a specific prey from numerous common items can introduce uncertainty into consumption estimates. Here, the tailor prey with the highest energy density (7.06 kJ g^−1^) was 268% greater than the prey with the minimum energy density (2.63 kJ g^−1^). The energy density of prey items is a particularly important parameter in bioenergetics modelling because it acts as a scaler for the final consumption estimate (in grams of prey) produced by the model. If the value of energy density is under or overestimated by 50%, the final consumption estimate will also be under or overestimated by 50%. There are few other parameters in bioenergetics models with such a large impact on final consumption estimates. For example, the growth parameter may comprise up to 30% of total energy expenditure^28^, but an error in growth rate of 50% will only result in a 15% error in a consumption estimate. With recent advancements in electronic tagging technology, there is a current focus on gathering accurate field-based energetic data for use in bioenergetics models^29,30^. These advancements are important for learning about free-ranging animals and could improve the model presented here by accurately estimating activity costs rather than using a constant activity multiplier that assumes no difference in activity levels through ontogeny. However, models may poorly convert highly accurate energy requirements (in joules) to a useable prey consumption (in grams) if diet variation is not considered.

### How many prey types, how many stanzas

Not all models contain a resolved age- or size-structure, but all estimates of animal consumption (be it adult, lifetime, or size-structure consumption) will benefit from incorporating dietary ontogeny is some way. It will always be better to estimate consumption using averages of mass-weighted energy densities for multiple prey items, and this can be achieved in some cases with only a few of the most common prey species (Fig. 5A). Great benefit will also come from splitting a species into stanzas when possible^31,32^ to account for differences in juvenile and adult prey composition, and, depending on the pattern of changing prey composition, a great improvement in accuracy of composition may be achieved with only 2–3 stanzas (Fig. 5C). This is especially true for juveniles, which can have vastly different diets to adults, and improving the accuracy of juvenile consumption then benefits the accuracy of lifetime mean consumption estimates.

Ecosystem models rely on consumption rates; for example, in the Ecopath with Ecosim framework^33,34^ biomass flux among trophic groups is defined by each group’s Q:B. Ontogenetic differences are of course acknowledged in these types of models, as are the challenges in representing all trophic impacts from diet ontogeny^35^. Although using a single Q:B value for a species or group is most common, a single group can also be split into stanzas. But even if a species is split into stanzas, the Q:B values for the different stanzas are necessarily based only on a standard allometric scaling relationship^34^. Given the potential influence of ontogenetic diet variation on Q:B, we recommend more emphasis be placed on determining prey energy densities to inform the Q:B values for groups and stanzas in ecosystem models. Even if a model is structured so that there must be a single Q:B value for a species or group, this can still be derived from a bioenergetics model that accounts for diet ontogeny by calculating a biomass-weighted Q:B from a size- or age-based bioenergetics model (e.g. Fig. 4).

The degree to which consumption estimates can be improved by using multiple prey items and stanzas will vary between species and likely depends on two factors. First, the number of prey items required to account for a sufficient amount of the variation in prey energy density will depend on the diversity of a predator’s diet; e.g. for generalist predators more prey items will need to be sampled to provide an accurate estimate of mean energy density, compared to consumers with specialist diets. For tailor in this study, reasonable estimates of prey energy density are achieved with ~5 prey items (Fig. 5A), which made up 65% of the total diet diversity (by mass; Fig. 5B). Species with more specialised diets (and less variability in energy density) will need fewer prey items to account for similar proportions; e.g. copepods alone comprise 87% of the diet of anchovy *Engraulis encrasicolus*^36^, so it would likely be sufficient for anchovy to measure only the energy densities of representative copepod species. Second, splitting consumption into multiple stanzas will improve consumption estimates most for species with high ontogenetic diet variation, particularly where diet shifts are observed (e.g. shifting from invertebrates to fish in the present study). Here, the Q:B of simulations improved when consumption was calculated from three stanzas, which aligns with diet information on tailor whereby diet shifts cause three ‘clusters’ of prey types^27^. Diet shifts are commonly seen in marine predators^22,37,38^, but this is species-specific and the appropriate number of stanzas useful for estimating consumption will vary between species.

The combination of diet diversity and ontogenetic diet shifts will determine the importance of considering a model like our full model presented here. For example, Australian bass *Macquaria novemaculeata* have high diet diversity throughout life but no major diet shifts^17^, mulloway *Argyrosomus japonicus* have moderate diet diversity but experience a strong diet shift from mysid shrimp to fish^37^, and tailor exhibit both high diet diversity throughout life and two ontogenetic diet shifts^27^. Similarly, the importance of accurate consumption estimates may need to be emphasised under certain research questions, e.g. contaminate uptake or changes in prey/habitat availability. Regardless, using multiple prey types in multiple stanzas should be the starting point for calculating prey energy densities when estimating consumption.

Interestingly, we saw Q:B improve incrementally as consumption was calculated from more stanzas, however total consumption by mass showed a less clear trend. Total consumption was less accurate when calculated from the three-stanza simulation than the two-stanza simulation. This is because the prey energy density using two stanzas was more accurate for large juveniles, which account for most of the consumption (by mass) in the first year. Conversely, using three stanzas gives a better fit of the mean consumption rate for juveniles, and hence the mean Q:B will be more accurate. As a result, these two metrics provide different methods of measuring error. If measuring total consumption by an individual fish is the primary concern, then the prey energy density used should aim to be closest to the life stage where the highest absolute consumption occurs. However if population level consumption is required, an accurate Q:B should be emphasised, because although juveniles have a smaller individual consumption by mass, they have the highest abundance and total population consumption can peak in the juvenile phase^39,40^.

### Juvenile diet and consumption rates

The juvenile phase is usually when the greatest change in size, speed, morphology, hunting ability, and prey selection occurs in predators^19,37,41^. Population consumption estimates are often based on adult consumption rates^42–45^ and are thus likely to be underestimating consumption by juveniles. The simulation presented here illustrates the impact of juvenile predators on the consumption of prey, with juvenile tailor in the estuary consuming at least 3–10 times that of adults per unit biomass. This heightened consumption rate of juveniles is a result of consuming less energy-dense prey items, as well as the effect of body mass on metabolic rate, and increased energy requirements associated with more rapid growth rates^24,46^.

Including aspects of ontogeny in consumption estimates is particularly important for consumers with spatially separated life history stages, such as tailor. Consumption rate estimates that do not consider spatially explicit ontogenetic life history stages may result in errors in consumption rate estimates that are not evenly distributed throughout the consumer’s entire habitat, but rather will be focused on certain locations. For example, if juvenile tailor consumption rates are not accounted for in overall models of the species’ consumption, the errors will be primarily concentrated in estuaries where the juveniles are distributed. Because we know that adult tailor rarely enter estuaries^47^, we can quantify the consumption by tailor that is underestimated specifically in estuaries if ontogeny is not considered in models (i.e. the difference between juvenile and adult consumption rates).

### Future directions and conclusions

Including detailed ontogenetic diet and prey energy density information in biogenetics models can improve consumption estimates. Even if detailed prey information is lacking for a consumer, bioenergetics models could still provide multiple outputs of prey consumption corresponding to any available prey energy density data, as a way of communicating a possible range of prey consumption estimates. While prey energy density is an important parameter in estimating consumption from a modelling perspective, its real impact on free ranging animals should not be overlooked. For example, yellowfin tuna *Thunnus albacares* need to consume 66% more prey to maintain growth rates if feeding solely on cephalopods rather than fish^48^, which, in a world of increasing cephalopod abundance^49^, is important for understanding the trophodynamics of such a commercially valuable species. Similarly, potential changes to prey items should be considered with respect to climate change^50,51^, as many range shifting fish species may move to areas with altered prey availability^52^.

Ecosystem based fisheries management is designed to ensure that human harvest of prey species does not negatively affect the sustainability of fish populations or compromise healthy ecosystem function^53,54^. To most effectively inform management, the consumption requirements of entire cohorts (animals of the same age) or populations are needed. Individual fish in the simulations presented here are representative of these cohorts, in that all other fish in the cohort will experience similar shifts in diet and Q:B with ontogeny. Consumption requirements, such as those calculated here, can be extrapolated to the population level if accurate biomass and mortality estimates are available. In this way, more realistic bioenergetics models that include ontogenetic variation in diet can lead to more informed and effective management of exploited fish populations. Hughes *et al*.^2^ estimated a 10,000 tonne population of Australian salmon in south-eastern Australia would consume 36,296–48,190 tonnes of prey annually; removing ~15% of the biomass of its main prey species. Using the error range presented here by using a single prey energy density, the cohort consumption estimate of Australian salmon becomes 29,557–60,313 tonnes (12–19% of prey biomass).

Other properties of prey items can have lesser impacts on consumption models, including the assimilation efficiency between different prey sources depending on nutritional composition^55,56^. Including this variation in bioenergetics models would result in the mean energy density being weighted not only by the dietary proportions of prey but also by their specific assimilation efficiencies. Most consumption models^2,28,57^ only present a mean assimilation value for each consumer (including our study, in parameter *A*). The assimilation efficiency of different prey items can be determined with feeding experiments^58^, with the greatest differences likely to occur between major food types (e.g. animals versus plants)^59,60^. It should be noted that a predator may be able to obtain a similar energy content from its diet while changing the proportions of macronutrients ingested^61^. Furthermore, different sources of the same macronutrient (e.g. carbohydrates) may represent differing levels of assimilation (e.g. starch vs cellulose) and this can impact energy ingestion^61^. Food assimilation in fish is generally considered to account for 5–20% of total energetic costs^62,63^, although it has been measured for tunas at 35%^64^. Therefore, variation in assimilation efficiency may represent an aspect of bioenergetics models that can also be improved to increase accuracy of consumption estimates.

Ontogenetic diet shifts may also include consumption of larger individuals within prey species, and energy density may increase with body size within species^65,66^. Therefore, the difference in energy density between the prey of juvenile and adult predators may be greater than presented here and would lead to greater differences in consumption estimates between models that include detailed ontogenetic prey energy density and those that don’t. Similarly, predator energy density may increase with size, meaning that the cost of growth may be lower or higher than expected for juveniles or adults, respectively. Accounting for size-dependent predator energy density may influence absolute consumption estimates for juveniles in bioenergetic models and is an avenue that could be explored in future studies.

Although the consumption rate estimates presented here were not validated using laboratory tests, we assume more detailed data would produce a model that more accurately represents the real world. Additionally, integrating data from our respirometer experiment further bounds our modelled consumption rates to reality. Our estimates of tailor consumption rates are similar to other similar predators in the ecosystem. Specifically, the mean adult tailor Q:B presented here (7.7) is intermediate compared to other local mesopredators; Australian salmon *Arripus trutta* (Q:B 3.2–5.252) and Australian bonito *Sarda australis* (15.6^67^). The similarities in these Q:B ratios are related to similarities in fish growth rates, activity levels, and morphologies. Australian salmon is similar to tailor in diet, size, behaviour and habitat, but is a slower growing species^68,69^ and Australian bonito is an active, medium-sized scombrid that is expected to have high energy requirements based on caudal aspect ratio^70^.

The energy density of prey, and how it changes throughout a consumer’s ontogeny, appears to be an underappreciated parameter in bioenergetics modelling. There are few parameters of a bioenergetics model to which an estimate of prey consumption would be more sensitive, possibly with the exceptions of allometric metabolic scaling and temperature (which both have exponential relationships with metabolism). Despite this, consumption models often overlook variation in prey energy density that arises from consumer ontogeny and habitats, primarily because this information if often lacking for marine consumers. Frequently studied species, such as those of commercial importance, may offer opportunities to further sample diet data if existing diet records are unavailable. In cases where detailed diet data is unavailable, we suggest considering juvenile and adult consumption separately, and to use what diet data is available to guide a prey composition that will be more representative of free-ranging animals than simply choosing a single, common prey item. We recommend developing species-specific versions of equation (6) presented here to incorporate ontogenetic diet and prey energy density in consumption models, especially for species with spatially segregated life stages and for species with variable diets, as the consumption estimates of these species are those most likely to improve with this additional trophic information.

## Materials and Methods

Tailor (*Pomatomus saltatrix* Linneaus 1766) are a globally distributed generalist predator^71^, with spatially segregated juveniles which may reside in estuaries for approximately one year before migrating to coastal waters as adults^72^. This ontogenetic migration is accompanied by a diet shift resulting from altered prey availability and increased foraging ability. Tailor can prey on a range of fish and invertebrates and a large amount of variation exists in their diet^27,73^.

### Measuring metabolic rate

Metabolic rates are the foundation for quantifying the energy requirements of consumers, and are usually measured using respirometry. We used two respirometry experiments to measure the effect of body mass and temperature on the resting mass-specific metabolic rate (RMR; g O_2_ g fish^−1^ d^−1^) of tailor to parameterise a bioenergetics model. The mass-dependent respirometry experiment determined the relationship between body mass and RMR at 24 °C using 24 tailor consisting of 16 juveniles (11–25 cm fork length (FL), 18–275 g) and 8 adults (33–42 cm FL, 500–1035 g). The temperature-dependent respirometry experiment tested the effect of temperature on RMR at 18, 21, 24, 27 and 30 °C (±1 °C), with 12 juvenile tailor in each temperature treatment (10–25 cm FL, mean mass 68.8 ± s.e.m. 6.0 g). Juveniles were used to allow the greatest breadth of trials; both in terms of temperature range and individuals within temperature treatments. We assumed juveniles and adults have the same response to temperature, and that any difference would not change the conclusions regarding differences in consumption between models with varying levels of prey energy density information.

Tailor were caught in Sydney Harbour, Australia, and transported to aquarium facilities at the Sydney Institute of Marine Science (SIMS), Chowder Bay (33°50′31.6″S; 151°14′51.18″E), and fed daily a diet of frozen fish, cephalopods, and crustaceans. Holding tanks were maintained at the treatment temperatures using bar heaters or an Oasis EC9bp water chiller (Oasis Heat Pumps Knoxfield, Victoria, Australia). Holding tanks were adjusted 1 °C each day until the treatment temperature was reached^74^, and held at this temperature for one week before experiments began^75^.

The RMR of individual tailor was determined by measuring oxygen consumption in darkened respirometry chambers. Rectangular chambers of varying size were used, with fish mass to water volume ratios (g:mL) between 1:20 and 1:100^76^. Prior to the respirometry trials, tailor were fasted for 24 hours and acclimated for 3 hours in the respirometry chamber^77,78^. The chamber was sealed from atmospheric air at the beginning of each respirometry trial. Oxygen concentration was measured by an oxygen meter (Hach HQ40d Loveland, Colorado, USA), and each respirometry trial ran until the dissolved oxygen concentration reached 80%. Microbial respiration in the respirometer was measured after each fish was removed from the chamber and subtracted from the total oxygen consumed to calculate oxygen consumption by the fish only^79^.

Two relationships were derived to describe the metabolic rate of tailor. First, the relationship between body mass (g) and the mass-specific RMR (gO_2_ g^−1^ d^−1^) of tailor was determined using linear regression of log-transformed values. This relationship was used to parameterise the bioenergetics model (*R*_*A*_ and *R*_*B*_ in equation 3 below) and was isolated from the effect of temperature on RMR (*R*_*Q*_ in equation 4 below), which was examined separately. Second, a linear regression of log-transformed values was used to determine the effect of temperature on the mass-specific RMR (gO_2_ g^−0.76^ d^−1^) of tailor. To isolate the effect of temperature and account for the effect of body mass on metabolic rate, a scaling exponent (0.76) was used in estimating the relationship between RMR and temperature. This scaling exponent was informed from the first, mass-dependent respirometry experiment and subsequent relationship between body mass and RMR. All statistical models were done using R statistical computing (v3.3.1; R Core Development Team 2016). All experiments were performed in accordance with relevant guidelines and regulations under approval by the University of New South Wales Animal Care and Ethics Committee (No. 15/152B).

### Estimating energy requirements

To demonstrate the difference in consumption (in grams) given a consumer’s ontogenetic diet variation, the energy requirements (in joules) of the consumer at any one time must first be quantified. The energy requirement (*C*; J d^−1^) of individual tailor was estimated using a bioenergetics model that incorporated the mass- and temperature-dependant metabolic rates determined from the above respirometry experiments. The bioenergetics model used was based on the energy balance model of Kitchell, *et al*.^28^ (Supplementary Note):

1 $$C=\frac{R+G}{1-A}\,$$

where *C* is energy requirement (J d^−1^), *R* is energy required for metabolism (J d^−1^), *G* is energy allocated to daily fish growth (J d^−1^), and *A* is the proportion of energy that is lost to food digestion, egestion, and excretion (J d^−1^; Table 2). Energy required for daily growth (*G*; J d^−1^) was determined by:

2 $$G=\Delta W\,Fj$$

where *ΔW* is the daily change in fish mass (g d^−1^) and *Fj* is the energy density of somatic tissue of tailor (J g^−1^; Table 2). Δ*W* (for an individual at a given weight) was calculated using the von Bertalanffy growth equation^69^ (Table 2), converted to mass from fish length using a length-weight relationship (Weight = 0.0104 Fork Length^3.0824^; H. T. Schilling, unpublished data). *Fj* was estimated from bomb calorimetry analysis (see details below). Spawning losses for adults were not accounted for in the simulations due to a lack of baseline information, however while their inclusion would benefit the model in general, it does not affect comparisons between our models.

**Table 2:** Summary of parameter mean values and standard deviations (s.d) used in the bioenergetics model.

Parameter description	Symbol	Value	s.d	Units	Equation	Source
Proportion of ingested energy lost to egestion, excretion, and digestion	*A*	0.319	0.068	—	1	Derived from Hartman and Brandt^5^
Energy density of *P*. *saltatrix*	*Fj*	7057.1	1316	J g^−1^	2	Measured
*Von Bertalanffy growth curve parameter	*t*_0_	−0.119	0.07	—	—	^69, 71^
Von Bertalanffy growth curve parameter	*k*	0.31	0.015	—	—	^69^
Von Bertalanffy growth curve parameter	*L*_∞_	81.5	0.75	cm FL	—	^69^
Mass-dependent intercept of metabolic rate	*R*_*A*_	0.0047	0.0001	—	3	Derived
Mass-dependent gradient of metabolic rate	*R*_*B*_	−0.2406	0.047	—	3	Derived
Activity multiplier	*ACT*	1.881	0.502	—	3	^5^
Oxy-caloric coefficient	*oxy*	14140	0.135	J gO_2_^−1^	3	^80^
Temperature-dependent gradient of metabolic rate	*R*_*Q*_	0.091	0.009	—	4	Derived
Energy density function coefficient	*EW*_*A*_	1.293	—	—	6	Derived
Energy density function constant	*EW*_*B*_	1.361	—	—	6	Derived

*The von Bertalanffy growth equation was taken from USA *P*. *saltatrix*^69^, and the *t*_0_ parameter was modified here (originally *t*_0_ = −0.3) to better represent the juvenile phase of Australian *P*. *saltatrix* in the estuary^71^.

The energy required for metabolism (*R*; J d^−1^) was calculated, using parameters derived from our respirometry experiments, as:

3 $$R={R}_{A}({W}^{{R}_{B}})f(T)ACT\,oxy$$

where *R*_*A*_ and *R*_*B*_ are the intercept and slope, respectively, of the logged mass-dependant function of mass-specific RMR, *W* is age-specific fish mass (g), *f(T)* is a temperature-dependence function, *ACT* is a constant multiplier which accounts for active metabolic rate, and *oxy* is the caloric coefficient of oxygen (J gO_2_^−1^) which is commonly used in animal energetics^80^ to convert the units of metabolic rate from oxygen (gO_2_ d^−1^) to energy (J d^−1^; Table 2). *R*_*A*_ and *R*_*B*_ were derived from the mass-dependant laboratory respirometry experiment (Table 2). *W* was derived from the von Bertalanffy growth curve as detailed above. *ACT* accounted for the heightened energy use in active metabolic rate associated with periods of activity by the fish, and is a constant multiplier of *R*^5^. The temperature-dependence function (*f(T)*) accounted for variation in metabolic rate due to changes in water temperature:

4 $$f(T)={e}^{{R}_{Q}(T)}$$

where *R*_*Q*_ is the slope of the logged temperature-dependant function of resting metabolic rate, and *T* is water temperature (°C). *R*_*Q*_ was derived from the temperature-dependant laboratory respirometry experiments (Table 2).

### Integrating ontogenetic diet information

To demonstrate the influence of including ontogenetic diet information in a bioenergetics model, we tracked the energy requirement (J d^−1^) of an individual tailor throughout its life and converted this to a consumption rate (g d^−1^) dependent on diet composition. A model was used to track the energy requirement (J d^−1^) through time of a typical tailor recruiting to an estuary: Sydney Harbour, Australia. As the above energy requirement (*C*) is temperature-dependent, temperature data from Sydney Harbour was used to estimate *C* for the model. Daily water temperature measured over 3 years (2013–2016) at Chowder Bay was used to generate a seasonal sine function of daily mean temperature for Sydney Harbour (range 15.6–22.1 °C). The primary spawning event for tailor occurs in late winter and spring^47^, and so the tailor was assumed to enter the estuary on the first day of spring (September 1).

The energy required by tailor (*C*, J d^−1^, equation (1)) was converted to a consumption rate of prey (g d^−1^), by dividing *C* by the mean energy density (*E*, J g prey^−1^) of the prey items. To demonstrate the importance of including consumer length-dependent prey energy densities in bioenergetics models, we required a relationship between *E* and tailor body length. This was done by calculating the mean energy density of prey (*E*) for every 1 cm tailor size class using detailed length-dependant tailor gut content data (FL 3–76 cm, n = 1437)^27^. The mean energy density of tailor prey for each tailor size class was calculated as the average prey energy density weighted by each prey’s proportional contribution (by mass) to the diet at that 1 cm size class:

5 $${E}_{x}=\sum _{1}^{n}P{E}_{i}\,P{P}_{i}$$

where *E*_*x*_ is the weighted mean energy density of prey at predator size *x* (J g^−1^), *PE*_*i*_ is the energy density of prey item *i* (J g^−1^), and *PP*_*i*_ is the proportion of diet comprised by prey item *i* at size class *x*.

Bomb calorimetry (6400, Parr Instrument Company, Illinois, USA) was used to measure the energy density of the prey, and was done for ten common prey types found in tailor diets. These ten prey types were used alongside published sources to estimate the energy density of all prey items (Table 1). Samples of prey for bomb calorimetry were obtained from the Sydney Fish Markets (Sydney, Australia), which were sourced from commercial fisheries close to Sydney. Preparation of prey samples for bomb calorimetry was done as described by the “subsample method” outlined by Glover, *et al*.^81^, whereby whole fish samples are homogenized and a small subsample is burned to determine the mean energy density of the fish.

The mean energy density of prey (*E*_*x*_, J g^−1^) for the tailor size classes were fitted against tailor fork length using a logarithmic function to create a continuous relationship for use in the model:

6 $$E=E{W}_{A}\,\mathrm{ln}(FL)+E{W}_{B}$$

where *FL* is fork length of tailor (cm), and *EW*_*A*_ and *EW*_*B*_ are constants (Table 2). Using this equation, *E* estimates the mean energy density of prey which is typically consumed when tailor are a specific size. Equation (6) was used to determine *E* for tailor with FL ≤ 42 cm. However *E* was fixed at 6300 J g^−1^ for tailor with FL > 42 cm, as this is the maximum mean weighted energy density of prey items consumed by free ranging tailor.

The ‘consumption: biomass ratio’ (Q:B) metric was used to communicate how consumption estimates change when using high quality diet and energy density information. Q:B indicates annual consumption by an individual relative to its biomass, and is a common metric to indicate the trophic impact of a consumer^33,82^. Q:B was calculated by expressing consumption rate (g d^−1^) as a proportion of fish mass and multiplying by 365 days to reach annual consumption relative to body mass (g g^−1^ y^−1^).

To quantify the effect of integrating variable prey energy density with consumer ontogeny in the model, the consumption calculated from the full model (using consumer-length dependant *E* data, equation (6)) was compared with two alternative models. Each alternative model used only a single value for *E* derived from one of the two most common prey items for adult tailor (Australian sardine *Sardinops sagax* and Australian anchovy *Engraulis australis*). This approach of estimating *E* from only the most common prey types is typical of numerous bioenergetics models^2–4^. To compare between our model and these two alternative models, consumption was expressed as the Q:B at 1 and 4 years of age (Q:B stabilised at ~4 years), as well as the total juvenile and lifetime consumption (in grams) for an individual fish. The energy density value for sardine was derived from bomb calorimetry, and the value for anchovy was taken from published sources (Table 1).

### How many prey types, how many stanzas

Acknowledging that highly detailed size-structured diet data is not always available, we also performed two simulations to explore how incremental increases in dietary information can improve the accuracy of consumption estimates. The first simulation examined the number of most common prey items that should be included in a model to reach a reasonable estimate of the true energy density of prey consumed by tailor. This was done by adding prey items one by one, weighted by proportion in diet, for juvenile (<1 y) and adult (>1 y) fish separately, and comparing the resulting prey energy density to the true mean prey energy density consumed (i.e. the weighted mean of all prey items consumed by juveniles or adults). We also determined the percentage (by mass) of total diet that was accounted for as prey items were added sequentially for both juveniles and adults of our predatory fish.

The second simulation using less detailed diet information examined the effect of measuring lifetime consumption from multiple stanzas (life history stages), and compared results to our full model. We ran simulations that used one, two (juveniles and adults; 0–1 y, >1 y), or three (0–0.5 y, 0.5–1 y, >1 y) stanzas, with each stanza having a single value for prey energy density measured as the weighted mean of the five most common prey types of that stanza. Five prey items were used based on the results of the previous simulation that added prey items sequentially to compare resulting prey energy density with our full model. The resulting estimated consumption rates, measured as total consumption in grams and mean Q:B, were then compared to results from our full model where prey energy density was a function of predator size, for both juvenile (first year) and lifetime consumption. Both simulations testing the effect of adding stanzas and prey items sequentially were truncated at 40 cm FL, as inconsistent sampling of gut contents at lengths greater than this introduced variation in the data that was not indicative of actual consumption.

### Data availability

The datasets generated during and/or analysed during the current study are available from the corresponding author on reasonable request.

## Electronic supplementary material

Supplementary Material
