# Supplementary material for: The influence of ontogenetic diet variation on consumption rate estimates: a marine example
Source: Sci Rep. 2018 Jul 16;8:10725. doi: 10.1038/s41598-018-28479-7 (PMC6048066; doi:10.1038/s41598-018-28479-7)
Supplement: Supplementary file 1 — Supplementary Material [file 41598_2018_28479_MOESM1_ESM.pdf]

The influence of ontogenetic diet variation on consumption rate estimates: a marine example

Christopher L. Lawson*[1,2], Iain M. Suthers[1,2], James A. Smith[1,2], Hayden T. Schilling[1,2], John Stewart[3], Julian M. Hughes[3], Stephanie Brodie[1,2]

[1] Evolution and Ecology Research Centre, and School of Biological, Earth and Environmental Sciences, University of New South Wales, Sydney, NSW 2052, Australia

[2] Sydney Institute of Marine Science, Chowder Bay Road, Mosman, NSW 2088, Australia

[3] New South Wales Department of Primary Industries, Sydney Institute of Marine Science, Chowder Bay Road, Mosman, NSW 2088, Australia

*Corresponding author: c.lawson@unswalumni.com.

Supplementary Material

Supplementary Note: derivation of equation 1

Kitchell, et al. [28] originally displayed equation (1) as

(7) $C = R + G + A$

where *C* is consumption rate (J d^{-1}), *R* is energy required for metabolism (J d^{-1}), *G* is energy allocated to daily fish growth (J d^{-1}), and A is the proportion of energy that is lost as waste (J d^{-1}). *C* and *R* are as described in the methods here, however *A* was calculated by Kitchell et al. as:

(8) $A = F + U + SDA$

where *U* is excretion (J d^{-1}), *F* is egestion (J d^{-1}), and *SDA* is specific dynamic action (J d^{-1}). *F*, *U* and *SDA* are constant proportions of consumption and were estimated as:

(9) $F = FA\ (C)$

(10) $U = UA\ (C - F)$

(11) $SDA = S\ (C – F)$

where *FA* is the proportion of ingested energy lost to egestion, *UA* is the proportion of assimilated energy lost to excretion, and *S* is the proportion of assimilated energy lost to digestive costs. *FA*, *UA*, and *S* are specific to species, and here values calculated for tailor were taken from the literature [5].

From equations (8-10) it can be seen that equation (6) contains consumption *C* on both sides. To solve for *C*, the right hand side of equation (6) must be stated in terms of *F*, *FA*, *U*, *UA*, *SDA*, and *S* only (i.e. not *C*), as these parameters have a known numerical value. From equations (8, 9):

(12) $U = UA\ (C – (FA*C))$

Therefore

(13) $U = C*UA\ (1 - FA)$

Similarly, from equations (8, 10):

(14) $SDA = C*S\ (1\text{-}FA)$

Now, from equations (8, 12, 13), equation (6) can be described as:

$$C = R + G + (FA*C) + (C*UA(1\text{-}FA)) + (C*S(1\text{-}FA))$$

Therefore:

(15) $$C = \frac{(G + M)}{1 - (FA + UA(1 - FA) + S(1 - FA))}$$

And inserting parameter values specific to tailor from Hartman and Brandt [5] (*FA* = 0.104, *UA* = 0.068, *S* = 0.172), equation (14) becomes:

$$C = \frac{G + R}{1 - 0.31904}$$

as presented in equation (1).

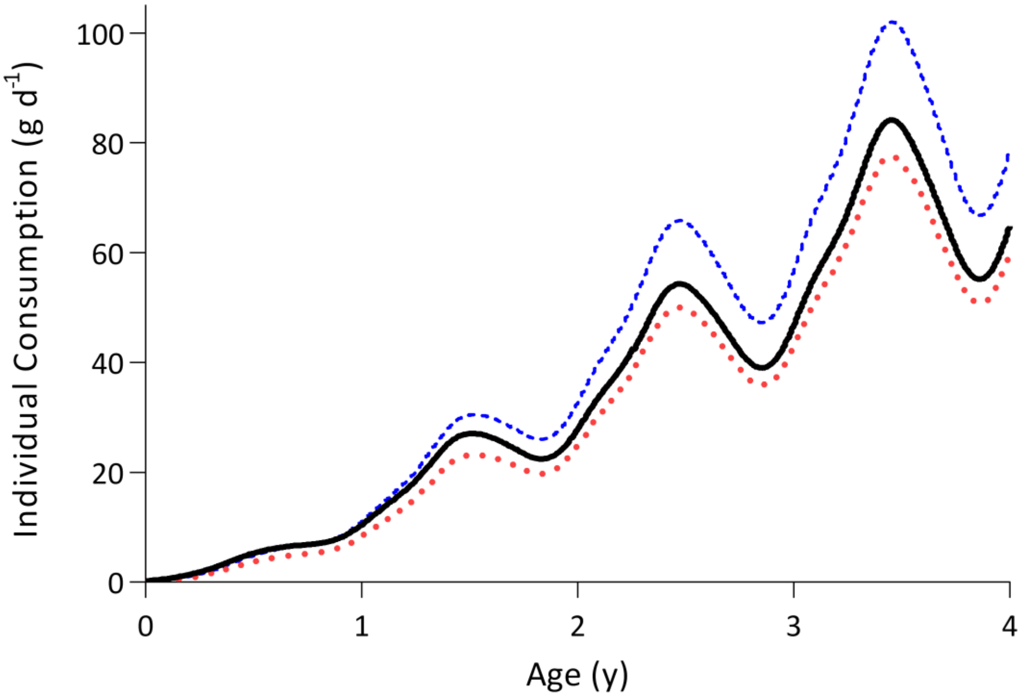

Fig. S1 – Daily individual consumption of tailor calculated using different prey compositions. Variable prey energy density (solid black), constant prey energy density based on a 100% sardine diet (high energy content; dotted red), and a constant prey energy density based on a 100% anchovy diet (low energy content; dashed blue). Peaks and troughs are driven by seasonal variation in water temperature.
